# *In Vitro* and *In Vivo* Antifungal Activity of Clove (*Eugenia caryophyllata*) and Pepper (*Piper nigrum* L.) Essential Oils and Functional Extracts Against *Fusarium oxysporum* and *Aspergillus niger* in Tomato (*Solanum lycopersicum* L.)

**DOI:** 10.1155/2020/1702037

**Published:** 2020-04-30

**Authors:** Laila Muñoz Castellanos, Nubia Amaya Olivas, Juan Ayala-Soto, Carmen Miriam De La O Contreras, Miriam Zermeño Ortega, Fabiola Sandoval Salas, Leon Hernández-Ochoa

**Affiliations:** ^1^Universidad Autónoma de Chihuahua, Facultad de Ciencias Químicas. Circuito Universitario, Campus Universitario No. #2, Chihuahua, Chihuahua C. P. 31125, Mexico; ^2^Tecnológico Nacional de México, Instituto Tecnológico Superior de Perote, Carretera Federal México-Perote 140, Centro, 91270 Perote, Veracruz, Mexico

## Abstract

In this study, hydrodistillation was used to obtain essential oils (EOs) from pepper (*Piper nigrum* L.) and clove (*Eugenia caryophyllata*) and co-hydrodistillation (addition of fatty acid ethyl esters as extraction co-solvents) was used to obtain functional extracts (FEs). Antifungal activity of EOs and FEs was evaluated by determination of minimum inhibitory concentration (MIC) against *Fusarium oxysporum* and *Aspergillus niger.* The results showed that pepper (*Piper nigrum*) and clove (*Eugenia caryophyllata*) essential oils and their functional extracts are effective *in vitro* at concentrations from 400 to 500 ppm after 10 days of culturing. The essential oils and functional extracts were used on tomato fruit samples at three different concentrations: 350, 400, and 450 ppm^5^. Clove essential oil reduced the growth of *Aspergillus niger* from 50% to 70% and *Fusarium oxysporum* to 40%. The functional extracts (FEs) of clove and pepper, mixed with ethyl decanoate (FEs-C_10_), were the best combination for protecting the tomato fruit in vivo against both phytopathogenic fungi. Gas chromatography-mass spectrometry (GC-MS) was used to identify eugenol as the principal compound in clove oil and limonene, sabinene, and *β*-caryophyllene in pepper oil.

## 1. Introduction

Tomato (*Solanum lycopersicum* L.) is the second most consumed vegetable worldwide [1]. Tomatoes are an important source of lycopene, carotenes, ascorbic acid, and potassium [2]. However, this crop is affected by several diseases caused by a wide number of pathogens, resulting in reduction in fruit yield and quality, coupled with large economic losses [1, 3]. Vascular wilt caused by *Fusarium oxysporum* is a serious soil disease of tomato [4]. The pathogen attacks susceptible plants by root infection and then clogs the vascular system with mycelia or spores causing vascular and leaf discoloration, often leading to the death of the plant [5, 6]. Likewise, *Aspergillus niger* is one of the major pathogenic fungi in tomato fruit, such as *Helminthosporium solani* and *Penicillium digitatum* [7] One of the most prominent alternatives to conventional agriculture is the use of plant products which have been shown to control many phytopathogens previously reported [7, 8]. Essential oils are one of these plant products with great potential for controlling plant diseases, since they present antibacterial, antiviral, antifungal, and insecticide activity [8, 9]. The antimicrobial activity of essential oils is directly correlated with the presence of their bioactive volatile components such as terpene compound (mono-, sesqui-, and diterpene), alcohols, acids, esters, epoxides, aldehydes, ketones, amines, ethers and phenols [9, 10]. The antifungal action of essential oils may be attributed to their capability to disrupt cell wall and cell membrane and coagulate the cytoplasm, and hence they damage cellular organelles and allow escape of macromolecules [11–13]. The lipophilic nature of EOs allow them to pass through the cell wall and damage the cytoplasmic membrane while disrupting various layers of polysaccharide, fatty acids, and phospholipids, eventually making them permeable [14–16]. Recently, antifungal mechanism of action of EOs has been reported by measuring the ergosterol content of plasma membrane of test fungi [17]. Ergosterol is a major sterol component of the fungal cell membrane responsible for maintaining the cell function and integrity. Ergosterol is found almost exclusively in fungi and therefore used extensively as an indicator of fungal biomass [18]. The most common method of essential oil extraction is by hydrodistillation, although petroleum-derived solvents are also used to purify plant-derived compounds. However, the latter method can be dangerous because of the use of explosive and toxic solvents. An option would be the use of alternative solvents, also known as green solvents because they are not derived from petroleum products; therefore, they are not as detrimental to the environment. Among those alternative solvents, fatty acid ethyl esters extracted from plants are becoming an important option because of their amphipathic property and biodegradability, and they are also nontoxic and nonirritable [19]. The aim of the present work was to evaluate the antifungal effects of essential oils and functional extracts from clove (*Eugenia caryophyllata*) and pepper (*Piper nigrum* L.) for reducing the severity of *Fusarium oxysporum* and *Aspergillus niger* wilt in tomato (*Solanum lycopersicum* L.) using a series of *in vitro* and *in vivo* evaluations.

## 2. Materials and Methods

### 2.1. Raw Material

The vegetal material used in this study was Clove (*Eugenia caryophyllata*) and pepper (*Piper nigrum L.*) provided by Comercial Cordona from Chihuahua, Mexico. The fatty acid ethyl esters used as co-solvent in the co-hydrodistillation process were ethyl decanoate (C_10,_ CAS number 110-38-3, 99%, Sigma-Aldrich), ethyl undecanoate (C_11,_ CAS number 627-90-7, 99%, Sigma-Aldrich), ethyl dodecanoate (C_12,_ CAS number 106-33-2, 99%, Sigma-Aldrich), and ethyl tetradecanoate (C_14,_ CAS number 124-06-1, 99%, Sigma-Aldrich). Isolates of *Fusarium oxysporum* and *Aspergillus niger* were used in this study. The strains are part of the Fungi Culture Collection of the School of Chemical Sciences.

### 2.2. Extraction of Essential Oils (EOs) and Functional Extracts (FEs)

Clove (*Eugenia caryophyllata*) and pepper (*Piper nigrum* L.) were individually subjected to a hydrodistillation process using the modified Schilcher device. In hydrodistillation, the plants were immersed in water, where the system was heated to the boiling point of water. For co-hydrodistillation, fatty acid ethyl esters were added as co-solvent, and the homogeneous mixture was called functional extracts (FEs). The fatty acid ethyl esters used in this study were ethyl decanoate (C_10_, CAS number 110-38-3), ethyl undecanoate (C_11,_ CAS number 627-90-7), ethyl dodecanoate (C_12,_ CAS number 106-33-2), and ethyl tetradecanoate E_14,_ CAS number 124-06-(1). For this process, 200 g of vegetal material, 4 L of water, and 20 mL of ethyl ester were used for the clove and pepper [20].

### 2.3. Analysis of Essential Oils and Functional Extracts

The essential oil and functional extracts were collected and diluted with hexane in a volumetric flask; the solutions obtained were analyzed by GC-MS (gas chromatography-mass spectrometry). The analysis of essential oils and functional extracts was conducted using a gas chromatographic system (Perkin-Elmer Instrument, Auto system XL, USA) equipped with a DB-5 column (5% phenyl methylpolysiloxane, 20 m × 0.1 mm i.d. and 0.4 µm film thickness) and a mass spectrometer (Perkin-Elmer instruments TurboMass Gold) as detector.. The carrier gas was helium, at a flow rate of 1 ml/min. For the GC-FID analysis, the temperature was increased from 60°C to 180°C at 1°C/min. The injector and detector temperature were set at 180°C.

#### 2.3.1. Effect of Essential Oils on Radial Growth: Minimum Inhibitory Concentration (MIC)

In order to prove the efficacy of the bioactive compounds of EOs and FEs against *F. oxysporum* and *A*. *niger,* the final concentrations of each used in potato dextrose agar (PDA) were 100, 200, 300, 400, and 500 ppm. The controls were prepared with agar and ethanol and pure ethyl decanoate, ethyl undecanoate, ethyl dodecanoate, and ethyl myristate at a 100 ppm concentration. Treatments were randomly arranged with four replications. To promote infection, treated tomato fruits were kept in a humid chamber at 30°C in darkness. The diameter of the radial growth caused by *Fusarium oxysporum* and Aspergillus niger was measured 10 days after inoculation.

### 2.4. Inoculum Production

The inoculum was prepared by culturing *Fusarium oxysporum* and *Aspergillus niger* in darkness at 25°C on PDA for 14 days in Petri dishes. The conidial suspension was prepared with sterile distilled water and Tween 80 and quantified by a Neubauer chamber to adjust at 1.5 × 10^5^ conidia/mL.

### 2.5. *In Vivo* Protection Assay

For the *in vivo* assays, three concentrations were tested: 350, 400, and 450 ppm, but only with the clove essential oil (EO), as well as different clove extracts (FEs) with different esters.

Fresh and healthy round tomatoes (*Solanum lycopersicum L*.) were disinfected with 90% ethanol and rinsed with sterile distilled water. A 6 mm diameter hole was made on the skin of the tomato fruit using a sterile cork-borer, and then 100 *μ*L of the spore suspensions (1.5 × 10^5^ conidia/mL) was added on the area; then the *epidermis* was put back and sealed with sterile petroleum jelly (only on the edges of the cut to avoid fruit oxidation). After fungal inoculation, tomato fruits were sprayed with EO and FEs at 350, 400, and 450 ppm. Control tomato fruits were sprayed with sterile distilled water-ethanol (2%). Treatments were randomly arranged with three replications. To promote infection, treated tomato fruits were kept in a humid chamber at 28°C in darkness. The diameter of the rotting area caused by *Fusarium oxysporum* and *Aspergillus niger* was measured every day during fifteen days' postinoculation.

## 3. Results and Discussion

### 3.1. Chemical Composition of Plant Essential Oils and Functional Extracts

The results obtained from the extraction process show that when comparing the extraction yields obtained by the hydrodistillation and co-hydrodistillation methods for each spice, the yield for clove is higher than pepper. This behavior is observed likewise in the percentages obtained from each of the functional extracts. This can be explained generally because in the plant, essential oils are stored or located in glands, conduits, sacs, or glandular hairs or in reservoirs in the plant; therefore, the exposure of these reservoirs to the action of the distillation vapor allows the vapor to soften or break the walls of the oily glands, releasing the oils, favoring extraction yield [11]. Results obtained from the chromatographic analysis are detailed in Table 1, which shows the principal components of clove and pepper essential oils and functional extracts. In the clove extracts, *Eugenol* was identified as majority component, which is also found in majority proportion (70–85%). Other determined components are the esters ethyl decanoate, ethyl undecanoate, ethyl dodecanoate, and ethyl tetradecanoate, whose percentage corresponds to 4–17% of the relative composition; these were added in the co-hydrodistillation process. In addition to these components, there were identified eugenyl acetate (15%) and *β*-caryophyllene. These results match Hernández-Ochoa et al. [21] and Jirovetz et al. [22], who report that clove essential oil may contain up to 90% eugenol, in addition to eugenyl acetate (2% to 7%), as well as the presence of caryophyllene. In the case of the pepper essential oil, it was determined the presence of limonene (18.8%), sabinene (16.5%), *β*-caryophyllene (15.6%), and *β*-pinene (10%) as majority compounds; in addition to this, another group of compounds was determined at a lower proportion (Table 2). These results are consistent with the reports of several authors [23, 24], where these are identified as majority compounds; however, they mention the presence of a total of 22 compounds in some cases, which differ from the total 8 compounds that were determined at a lower proportion. This difference in the chemical composition may be attributed to the origin of the fruit, as well as to different growing and/or simple conservation conditions.

### 3.2. Fungi Identification

**Figure 1:**
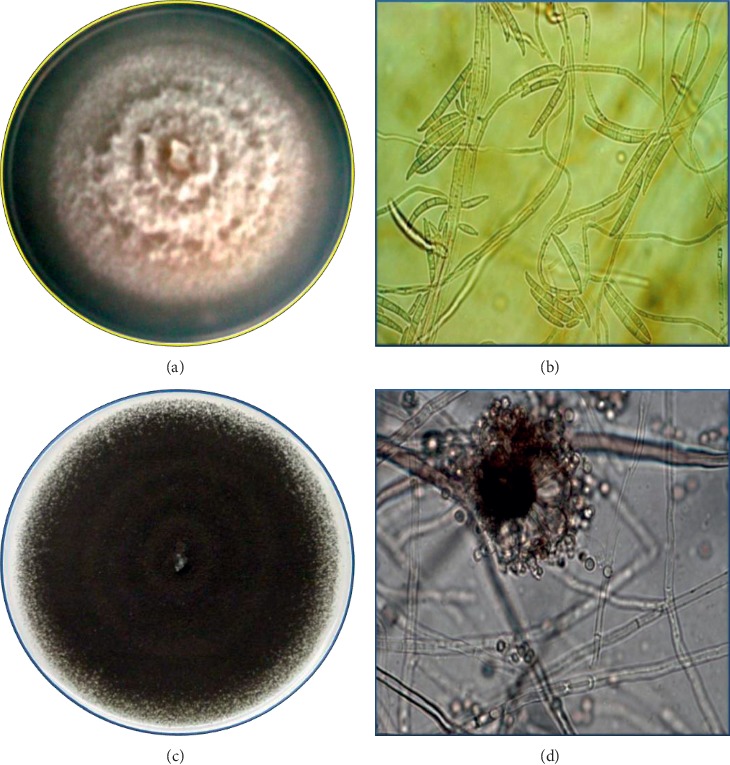
Colonies and microscopic morphology at 10x and 40x of *Fusarium oxysporum* (a, b) and *Aspergillus niger* (c, d) at 9 days of incubation on PDA agar.

*Fusarium oxysporum* showed an exponential growth, as well as a flat cottony morphology that tended to extend, developing a white aerial mycelium as observed in Figure 1. It can be observed the production of a mycelium of characteristic white color which turned into pink salmon; it was also observed that white colonies spread pigmentation to the medium. After 72 hours, the growth of the colonies was 2 cm. Microscopically were observed abundant microconidia, which are generated in simple phialides emerging laterally; they present a slightly curved oval-elipsoid form, and they lack septa. Macroconidia also present a pedicelled base; some have three to five septa; three-septate spores were more common. These results match the research of Nelson [25], where it is mentioned the presence of three kinds of spores: macroconidia, elongated spores with 3 to 5 septa, with a shape typical of the genus; microconidia, oval shaped and abundant; and chlamydospores that allow distinguishing *F. oxysporum* from *F. moniliforme* [10]. In the case of *Aspergillus niger,* it was observed that colonies, initially white, grew fast; however, the mycelial surface was covered with black spots that represent Aspergillus heads (Figure 1), measuring up to 1 mm in diameter, charged with black spores that turned into a pulverulent culture, forming a dense felt of erect conidiophores. The observed microscopic characteristics showed that it presents a single conidiophore originated in a partitioned hypha; this aerial conidiophore ends in an enlarged vesicle containing double or single projections; these are phialides, out of which extends a single, sometimes long chain of small round conidia [26].

### 3.3. Evaluation of *In Vitro* Antifungal Activity of EOs and FEs

#### 3.3.1. Antifungal Activity of Clove *(Eugenia caryophyllata)*

The extracts' *in vitro* fungicide activity was evaluated by determining the capacity of inhibition of mycelial growth of each of the species under study. The clove essential oil presented the greater biological activity of all the treatments used, achieving a total inhibition of the growth of the species evaluated. In the treatment of clove essential oil against *Fusarium oxysporum*, the biological activity was proportional to the increase in concentration, as shown in Table 3. It is observed a mycelial growth of 5.6 cm at a concentration of 100 ppm, with mycelial growth significantly reduced to 400 ppm (0.44 cm). At 500 ppm, it is observed a total inhibition of *F. oxysporum*, with the essential oil showing greater inhibition compared to the growth of the corresponding control sample at 7.72 cm. The same behavior was observed against the species *Aspergillus niger*; the results obtained show a mycelial growth of 6.12 at a concentration of 100 ppm, which is modified at 200 ppm obtaining a value of 1.98 cm; total inhibition is observed at 400 ppm and above. In the case of clove functional extracts against mycelial growth of *F. oxysporum* (Table 3), the same behavior of the essential oil was observed, that is, an average growth of 6.5 cm was observed at a concentration of 100 ppm. The extracts of clove-C_10_ and clove-C_12_ showed a mycelial growth for *A. niger* of 6.23 cm and 6.38 cm, respectively, at 100 ppm, very similar to the growth obtained at the same concentration of the clove essential oil. In the case of the extracts of clove C_11_ and C_14_ it was observed a mycelial growth similar to *A. niger* at 100 ppm of 7.17–7.19 cm, higher values than those obtained with the clove essential oil. Different studies have demonstrated that clove essential oil possesses *in vitro* antifungal activity, affecting species that often develop in food, such as *Paecilomyces, Penicillium* sp., *Rhizopus* sp*., Rhizomucor* sp., and even some species of *Aspergillus* [27]. It has also been demonstrated *in vitro* that clove inhibits the growth of *Aspergillus flavus, A. niger*, *Fusarium oxysporum*, *F. chrysogenum*, and *Penicillium* sp., starting at 500 and 1000 ppm [28]. Generally, the main components in essential oils reflect their biophysical and biological characteristics [9]. However, it is reasonable to assume that the biological activity of the clove essential oil can be related to the presence of a high concentration of *Eugenol* (75–100%) [29]. Components with phenolic structure such as eugenol are highly active against microorganisms [30]. This phenolic compound can denature proteins, and it reacts with phospholipids of the cell membrane that change their permeability [31], which is explained by the acid nature of the hydroxyl group, which forms a hydrogen bond with an active enzymatic core.

#### 3.3.2. Antifungal Activity of Pepper *(Piper nigrum)*

Pepper essential oils and functional extracts did not show a significant inhibition activity against *Fusarium oxysporum* and *Aspergillus niger.* In the case of the essential oil, it was observed a growth of 7.5 cm at 100 ppm and of 7.3 cm at 200 ppm, confirming that it remains constant up to 500 ppm where it is observed a reduction to 6.0 cm. In all the pepper functional extracts, there was a very similar behavior to essential oil, since in the extracts it was observed an average growth of 7.3 cm at concentrations of 100, 200, and 300 ppm, while at 400 and 500 ppm, there was a reduction to 6.8 cm and 6.5 cm, respectively. In the case of *Aspergillus niger*, the growth was not affected significantly by the pepper essential oil, which at the concentrations of 100–300 ppm remained between 7.33–7.36 cm; however, at 400 ppm, there was a slight decrease to 6.81 cm. In the functional extracts, it is observed a similar behavior, obtaining lower growth in the extract pepper-C_14_ of 7.25 cm and a maximum mycelial growth of 7.7 cm after the incubation period. Therefore, it may be concluded that pepper did not show fungistatic and fungicide activity before both fungi. However, studies have shown that pepper essential oil presents antifungal activity particularly against fungi such as *Trichophyton terrestre, T. tonsurans, Candida albicans, Monosporium apiospermum, A. fumigatus, A. nidulans, Sporotrichum schencki,* and *Histoplasma capsulatum* [32]; this antimicrobial activity of the essential oil is influenced by their chemical composition, constituted by a combination of monoterpenoids and sesquiterpenoids considered responsible of the activity against organisms [33]. Some authors [34, 35] mention that the primary target of antimicotic chemotherapeutic agents is the fungus membrane. Most fungi contain ergosterol as the main component of the membrane, except for some oomycetes. Thus, many antimicotic agents act either fixing ergosterol or blocking biosynthesis, inhibiting an enzyme of the group cytochromes P-450 which catalyzes one of the first steps in esterol biosynthesis in fungi and animal cells, producingdemethylation of lanosterol, biosynthetic precursor of cholesteroland ergosterol. Others antimycotic agents block squalene epoxidase and are often used as topicals.Therefore, strategies that alter or block ergosterol synthesis affect permeability of cell membrane and the activity of enzymes linked to such membrane, which leads to inhibition of growth and later to cellular death.

### 3.4. Evaluation of *In Vivo* Antifungal Activity of EOs and FEs

Based on the results obtained in the *in vitro* tests, it was evaluated the biological activity of essential oils and functional extracts against *F. oxysporum* and *A. niger* with *in vivo* methods. The damage in the tomato fruit was analyzed. In the evaluation, the extracts with the higher inhibitory activity were used, that is, the clove essential oil and functional extracts: clove-C_10_, clove-C_11_, clove-C_12_, and clove-C_14_, at concentrations of 350 ppm, 400 ppm, and 450 ppm. The results obtained are shown in Table 4, where it can be observed that on the tomato surface there is a circular area, white at the beginning, evolving to a darker color. The lesions are observed covered with a cottony cluster of white mycelium, which causes softening and destruction of vegetable tissue; no exudate is produced, but dehydration and destruction of tissue occurs. As it can be observed, it was not possible to determine whether the clove essential oil and some functional extracts were capable of inhibiting the growth of *F. oxysporum*, except for the extract clove-C_10_ where it could be observed a significant variation in each treatment. Soft rot generated by *A. niger* could also be observed in the treatments where no inhibition was determined, with the presence of a fungal mass; in addition to this, no fungal development was present in the fruit surface, causing necrosis of the tissue that develops from the inoculation site to the internal region. Therefore, we can confirm that the essential oils and functional extracts evaluated *in vivo* against the species *A. niger* and *F. oxysporum* in the tomato fruit did not show strong inhibition; however, total inhibition could be demonstrated in the *in vitro* tests; this can be attributed to the existence of some factors that may affect the antifungal efficiency, such as the food components, inactivation due to the addition of other component, effects of pH over antifungal stability and activity, unequal distribution in the food matrix, and low solubility of extracts [36]. Additionally, Aguilar-Gonzalez et al. [37] demonstrated in vitro the effectiveness of mustard EO vapor against *A. niger* in tomatoes and concluded that mustard EO contains highly volatile compounds with strong inhibitory effects. However, these results were obtained with essential oils used in the vapor phase.

## 4. Conclusions

In conclusion, clove (*Eugenia caryophyllata*) and pepper (*Piper nigrum* L.) essentials oils and functional extracts could provide an alternative solution to the use of hazardous chemical fungicides for the treatment of tomato fruit during storage and transportation to reduce postharvest infection. In view of the economic importance of tomato, the possibility of using products with a lower risk to human health and the environment than synthetic pesticides in controlling *Fusarium oxysporum* and *Aspergillus niger* wilt is very promising.

## Figures and Tables

**Table 1:** Main compounds determined in clove (*Eugenia caryophyllata*) essential oil.

Compound	Retention time	Molecular weight	Molecular formula	Composition (%)
Eugenol	18.25	164	C_10_H_12_O_2_	85
*β*-Caryophyllene	19.58	204	C_15_H_24_	1.5
Eugenyl acetate	21.26	206	C_12_H_14_O_3_	5.5

**Table 2:** Chemical composition of pepper (*Piper nigrum*) essential oil.

Compound^(1)^	KI^(2)^	Molecular weight	Molecular formula	Composition (%)
*α*-Thujene	931	136.23	C_10_H_16_	1.4
*α*-Pinene	939	136.23	C_10_H_16_	5.7
Sabinene	976	136.23	C_10_H_16_	16.5
Β-Pinene	980	136.24	C_10_H_16_	10.7
Myrcene	991	136.23	C_10_H_16_	2.0
Limonene	1031	136.24	C_10_H_16_	18.8
*Β*-Phellandrene	1053	136.23	C_10_H_16_	1.8
Linalool	1 098	154.25	C_10_H_18_O	1.1
Terpinen-4-ol	1178	154.25	C_10_H_18_O	1.7
Δ-Elemene	1339	204.35	C_15_H_24_	1.9
Germacrene B	1560	204.35	C_15_H_24_	1.4
*β*-Caryophyllene	1625	222.37	C_15_H_26_O	15.1

^1^Main compounds in essential oil.^2^Kováts indices in reference to *n-*alkanes (C_8_-C_22_).

**Table 3:** Effects of essential oils (EOs) and functional extracts (FEs) of clove and pepper *in vitro* at various concentrations on radial growth of *Fusarium oxysporum* and *Aspergillus niger*.

	Clove (*Eugenia caryophyllata*)										Pepper (*Piper nigrum* L.)									
	*Fusarium oxysporum*					*Aspergillus niger*					*Fusarium oxysporum*					*Aspergillus niger*				
(ppm)	EO	FEs-C10	FEs-C11	FEs-C12	FEs-C14	EO	FEs-C10	FEs-C11	FEs-C12	FEs-C14	EO	FEs-C10	FEs-C11	FEs-C12	FEs-C14	EO	FEs-C10	FEs-C11	FEs-C12	FEs-C14
0	7.72	7.72	7.72	7.72	7.72	7.23	7.23	7.23	7.23	7.23	7.72	7.72	7.72	7.72	7.72	7.23	7.23	7.23	7.23	7.23
100	5.69	6.53	6.61	4.76	6.33	6.12	6.23	7.19	6.38	7.16	7.53	7.33	7.65	7.41	7.56	7.36	7.64	7.48	7.73	7.79
200	3.32	4.28	4.36	4.39	4.08	1.98	2.5	2.72	2.78	4.35	7.33	7.45	7.5	7.38	7.61	7.34	7.7	7.48	7.73	7.69
300	2.41	2.65	2.66	2.65	2.67	0.83	1.26	1.05	1.58	1.71	6.97	7.28	7.35	6.96	7.28	7.33	7.58	7.4	7.6	7.58
400	1.8	1.28	1.33	1.19	1.14	0	0.58	0.68	0.35	0.53	6.32	6.8	6.7	6.62	7.21	6.81	7.53	7.4	7.33	7.43
500	0	0.7	0.65	0.69	0.26	0	0	0	0	0	6.03	6.54	6.35	6.41	6.61	6.78	7.38	7.41	7.49	7.25

**Table 4:** *In vivo* antifungal activity of clove essential oil and functional extracts at *Solanum lycopersicum* L var “bola”.

	*Fusarium oxysporum*				*Aspergillus niger*			
EO/FEs	350 ppm	400 ppm	450 ppm	Control	350 ppm	400 ppm	450 ppm	Control
Clove EO	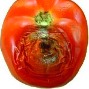	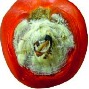	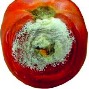	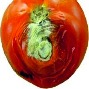	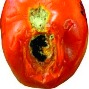	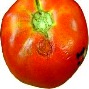	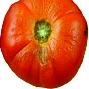	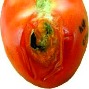
FEs-C_10_	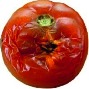	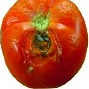	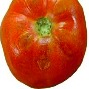	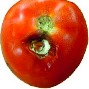	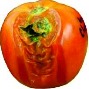	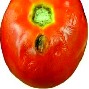	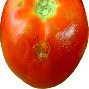	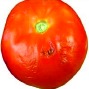
FEs-C_11_	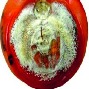	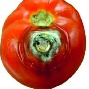	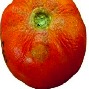	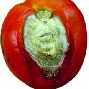	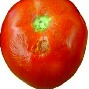	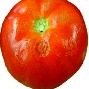	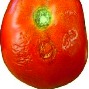	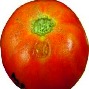
FEs-C_12_	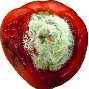	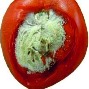	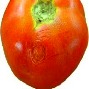	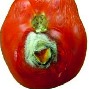	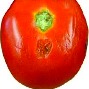	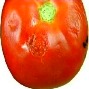	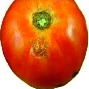	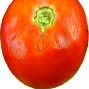
FEs-C_14_	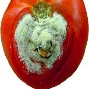	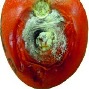	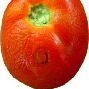	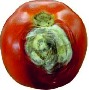	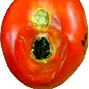	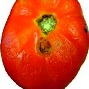	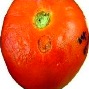	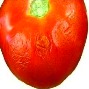

## Data Availability

The data used to support the findings of this study are included within the article.
